# Traditional Chinese Medicine Pien-Tze-Huang Inhibits Colorectal Cancer Growth and Immune Evasion by Reducing β-catenin Transcriptional Activity and PD-L1 Expression

**DOI:** 10.3389/fphar.2022.828440

**Published:** 2022-02-03

**Authors:** Qiang Chen, Yilin Hong, Shihe Weng, Peng Guo, Bei Li, Yong Zhang, Chundong Yu, Shicong Wang, Pingli Mo

**Affiliations:** ^1^ State Key Laboratory of Cellular Stress Biology, Innovation Center for Cell Biology, School of Life Sciences, Xiamen University, Xiamen, China; ^2^ Fujian Pien Tze Huang Enterprise Key Laboratory of Natural Medicine Research and Development, Zhangzhou, China

**Keywords:** pharmacology, colorectal cancer, immune evasion, PD-L1, pien tze huang

## Abstract

Pien Tze Huang (PZH) is a valuable traditional Chinese medicine, which has a variety of biological activities such as clearing heat-toxin, resolving blood stasis, detoxifying, relieving pain, and anti-inflammation. PZH has a partial role in suppressing the progression of CRC, while the underlying mechanism is a pending mystery; especially whether PZH mediates the immune escape of CRC remains unclear. Our study reported that PZH suppressed the proliferative activity of CRC by inhibiting Wnt/β-catenin signaling to down-regulate the expression of PCNA and Cyclin D1. In addition, PZH suppressed the immune escape of CRC and elevated the infiltration of CD8^+^ T cells in tumor tissues, which depends on the suppression of PD-L1 levels via inhibiting IFNGR1-JAK1-STAT3-IRF1 signaling. More importantly, PZH pharmacologically elevated the antitumor efficacy of anti-PD-1/PD-L1 immunotherapy as demonstrated by slower tumor growth, higher infiltration and function of CD8^+^ T cells in the combination of PZH and PD-1/PD-L1 antibody compared with monotherapy with either agent. These results demonstrate that PZH has the potential role in inhibiting CRC proliferation and immune evasion, especially the synergistic enhancement effect of PZH on immunotherapy.

## Introduction

Colorectal cancer (CRC) is one of the major threats to human health, and the traditional clinical methods are limited in the advanced stage of CRC (Dekker et al., 2019). Tumor progression is a complex process, involving the interaction between tumor cells and the immune system. Specific immunity mediated by immune cells is an important means to eliminate abnormal cell proliferation, in which CD8^+^ T cells play a vital role (Lim et al., 2016; Qian et al., 2018). However, cancer cells can produce certain key modifications through genetic or epigenetic changes to escape the surveillance of immune system and limit the efficacy of immunotherapy, including down-regulation of their major histocompatibility complex I (MHC I) antigen and up-regulation of PD-L1 inhibitory molecules (Vesely et al., 2011). Interferon γ (IFNγ), an important inflammatory factor released by effector T cells in the process of killing tumors, triggers cancer cell immunosuppression against CD8^+^ T cells surveillance via activating JAK-STAT pathway to induce the up-regulation of PD-L1 (Garcia-Diaz et al., 2017). Despite the tumor immunotherapy (e.g. Anti-PD-1/PD-L1 immune checkpoint inhibitor) has benefited cancer patients to a certain extent, it still has great limitations, such as insensitivity, poor prognosis and side effects (Johnson et al., 2016; Moslehi et al., 2018). Therefore, further enhancing the efficacy of immune checkpoint inhibitors is still one of the major issues to be resolved urgently.

Pien Tze Huang (PZH), a well-known and precious Chinese patent medicine, is composed of a variety of precious traditional Chinese medicines, including *Radix et Rhizoma Notoginseng* (Sanqi in Chinese, 85%), *Moschus* (Shexiang in Chinese, 3%), *Calculus Bovis* (Niuhuang in Chinese, 5%), and *Snake Gall* (Shedan in Chinese, 7%), which together have functions of clearing heat-toxin, resolving blood stasis, detoxifying, relieving pain, and anti-inflammation (Huang et al., 2019), and especially has remarkable hepatoprotective activities (Chan et al., 2004; Yang et al., 2018; Zheng et al., 2019). Many studies have shown that PZH has certain pharmacological effects in anti-tumor, such as CRC, liver cancer, breast cancer, and osteosarcoma (Huang et al., 2019). Zhuang and colleagues reported that PZH could inhibit CRC cells proliferation and promote their apoptosis via suppressing the STAT3 pathway (Zhuang et al., 2012). Shen et al. also reported that PZH up-regulated SOCS3 expression to suppress IL-6-induced STAT3 activation in human colon carcinoma cells (Shen et al., 2012); and the expression of proliferation-related genes, such as CDK4, p53, and c-Myc, was attenuated in PZH-treated HCT-8 cells (Shen et al., 2012; Wan et al., 2017). However, it is still unclear whether PZH inhibits the tumor immune escape by down-regulating the expression of immunosuppressive molecules in CRC cells.

In recent years, PD-1/PD-L1 immunotherapy combined with radiotherapy, chemotherapy, and molecular targeted therapy has benefited some people who are not suitable for PD-1/PD-L1 inhibitors (Keir et al., 2008; Li et al., 2016); and the effective targeted therapeutic inhibitors for CRC are still lacking. Therefore, we speculated whether PZH contributes to the antitumor effect of CRC immunotherapy. In this study, we found that PZH inhibited the proliferation of CRC cells *in vitro*, and stalled the xenograft tumor growth *in vivo* partially through inhibiting IFNGR1-JAK1-STAT3-IRF1 signaling to down-regulate the expression of PD-L1. Importantly, PZH pharmacologically elevated the antitumor efficacy of anti-PD-1/PD-L1 immunotherapy in mouse xenograft tumor models. Our results will contribute to the application of PZH in antitumor therapy, especially in tumor immunotherapy.

## Materials and Methods

### Mice

6-to 8-week-old male C57BL/6 mice were obtained from the Laboratory Animal Center of Xiamen University. All mice were randomly divided into groups and fed under the specific pathogen-free conditions with a 12:12-h light/dark cycle and received a regular chow diet and water ad libitum. All animal experiments performed following the ethical guidelines were approved by the Laboratory Animal Center of Xiamen University.

### Cell Culture

The CMT93 and HCT116 cell lines used in this study were obtained from Cell Bank of Type Culture Collection of Chinese Academy of Sciences (Shanghai, China) and preserved in our laboratory. The cell lines were characterized by Genetic Testing Biotechnology Corporation (Suzhou, China) using short tandem repeat markers. Cells were cultured using DMEM or RPMI 1640 medium (SH30045.05 and SH30045.04, HyClone) supplemented with 10% FBS (P30-3302, PAN-Biotech) and 1% penicillin-streptomycin (15140122, Thermo). All cells were tested negative for mycoplasma contamination using the single-step PCR method and were maintained in a humidified chamber with 5% CO_2_ at 37°C.

### Mouse Lymphocyte Cells

Healthy C57BL/6 mice were sacrificed and the spleen was isolated. Lymphocyte cells were isolated from mouse spleen using lymphocyte isolation kit (P8860, Solarbio) according to the manufacturer’s protocol. Lymphocyte cells were treated with PZH for 24 h and then activated with Phorbol 12-myristate 13-acetate (PMA, 50 ng/ml; P8139, Sigam) and Ionomycin calcium salt (Ion, 1 μg/ml; I8800, Solarbio) for 6 h.

### Pien Tze Huang Solution

The Pien Tze Huang (PZH) in powder form was obtained from Zhangzhou Pien Tze Huang Pharmaceutical Co., Ltd. (Zhangzhou, China). PZH extract for treating cells was prepared by dissolving the powder in PBS, ultrasonic treatment at 70 Hz for 30 min, and then centrifugation at 800 *g*. PZH suspension for treating mice was prepared by dissolving the powder in PBS and used directly after an ultrasound.

### Tumor Growth in Mice

A total of 2 × 10^6^ CMT93 cells were subcutaneously inoculated into the dorsal flanks of randomly selected C57BL/6 mice. Tumor-bearing mice were randomly divided into groups according to the established plan, and the indicated dose of PZH or anti-PD-1/PD-L1 antibody treatment was performed from the seventh day after tumor inoculation. The body weight and tumor size of mice were recorded every 2 days, and the tumor volume was calculated according to the following formula: Volume = Length × Width^2^ × 0.52. PZH was given intragastrically at a low dose of 0.078 g/kg, a medium dose of 0.234 g/kg and a high dose of 0.672 g/kg every day, respectively. PD-1 (BE0273, Biocell) or PD-L1 antibody (BE0101, Biocell) was intraperitoneally injected with a dose of 100 μg every 2 days. DMSO and isotype IgG were used as controls. At the end of the experiment, the mice were sacrificed, and tumor tissues were harvested for weight measurement and further analyses.

### Cell Clone Formation

The CMT93 or HCT116 cells were seeded into a 6-well plate with 1,000 cells per well according to the established grouping, and each group contains three parallel wells. After the cells were stabilized, the indicated dose of PZH was used to treat CRC cells.

The cells were fixed with methanol for 15 min after the clone was visible, and then stained with crystal violet staining solution (1%) for 5 min. Wash with water until the background is clean and imaged using GE Image scanner III (GE, United States).

### Topflash Assays

For luciferase assays, cells were co-transfected with Topflash reporter, Renilla luciferase plasmid and/or β-catenin plasmid. Cells were lysed in Harvest buffer and luciferase activity was determined using the luciferase reporter assay system (E2820, Promega). Transfection efficiency was normalized by Renilla luciferase activity.

### Western Blot

Cell or tissue was lysed in RIPA lysis buffer (50 mM Tris-HCl pH 7.4, 1% Triton X-100, 150 mM NaCl, 1% sodium deoxycholate, 1 mM sodium orthovanadate, 0.1% SDS, 10 mM NaF, 5 mM EDTA, 1 mM PMSF, 1% phosphatase inhibitors) and ultrasonic for 10 s using ultrasonic crusher at 20% power. Quantification of protein by BCA method and equal protein loading into SDS-PAGE gels and transferred into PVDF membranes (IPVH00010, Millipore), followed by immunoblotting with the specific antibody: GAPDH (AC033, ABclonal), β-actin (A5441, Sigma), H3-histone (ab1220, Abcam), p-STAT3 (ab76315, Abcam), STAT3 (9139S, CST), IRF1 (8478S, CST), mPD-L1 (60475, CST), hPD-L1 (13684, CST), IFNGR1 (PA5-96413, Thermo), JAK1 (50996, CST), p-JAK1 (74129, CST). Protein spots were imaged using a chemiluminescence imager (Tanon, China).

### Real-Time Quantitative PCR

Total RNA was extracted from tumor cells or tissue using TRIzol (15596018, Invitrogen) and was reverse-transcribed to cDNA using RT Master Mix (FSQ-201, Toyobo) according to the manufacturer’s protocol. The cDNA was subjected to quantification by real-time PCR using a CFX96 or CFX384 system (Biorad, United States) with qPCR Master Mix (AQ131-04, TransGen Biotech), and the reaction program is as follows: 98°C, 10 min; 98°C, 10 s, 60°C, 30 s, 40 cycles; 60°C, 5 s, 95°C, 10 s to set the melting curve. The relative expression level of gene mRNA was calculated according to the “2^^-△△Ct^” method and the β-actin was used as an internal control. The specific primers are as follows: m-β-actin, Forward 5′-ACT​ATT​GGC​AAC​GAG​CGG​TTC​C-3′, Reverse 5′-GGC​ATA​GAG​GTC​TTT​ACG​GAT​GTC​A-3′; m-PD-L1, Forward 5′-GTG​GTG​CGG​ACT​ACA​AGC​GAA​T-3′, Reverse 5′-ACG​GGT​TGG​TGG​TCA​CTG​TTT​G-3′; m-IRF1, Forward 5′-CGA​GGA​AGT​GAA​GGA​TCA​GAG​TAG​G-3′, Reverse 5′-GTG​GTG​TAA​CTG​CTG​TGG​TCA​TCA-3′; m-IFNγ, Forward 5′-ACT​CAA​GTG​GCA​TAG​ATG​TGG​AAG​A-3′, Reverse 5′-ATG​ACG​CTT​ATG​TTG​TTG​CTG​ATG​G-3′; m-Granzyme B, Forward 5′-AGA​ACA​GGA​GAA​GAC​CCA​GCA​AGT-3′, Reverse 5′-CCA​ACC​AGC​CAC​ATA​GCA​CAC​AT-3′; h-PD-L1, Forward 5′-TGG​CAT​TTG​CTG​AAC​GCA​TTT-3′, Reverse 5′-TGC​AGC​CAG​GTC​TAA​TTG​TTT​T-3′; h-IRF1, Forward 5′-GAT​GCT​TCC​ACC​TCT​CAC​CAA​GAA​C-3′, Reverse 5′-ATG​GCG​ACA​GTG​CTG​GAG​TCA-3′; h-β-actin, Forward 5′-CAT​GTA​CGT​TGC​TAT​CCA​GGC-3′, Reverse 5′-CTC​CTT​AAT​GTC​ACG​CAC​GAT-3′.

### Immunohistochemistry

Tissue with 4% formalin fixation and their chips with paraffin embedding were cut at 5 μm, followed by immunohistochemistry (IHC) staining according to directions of IHC kit (SP9000, ZSGB-BIO) and were incubated with CD3 (99940S, CST) or CD8 (98941S, CST) primary antibodies and biotinylated secondary antibodies, respectively. The signal was detected using HRP conjugated streptavidin with the chromogenic substrate DAB. The results were imaged by Leica DM4B Imaging system and interpreted by a senior pathologist. The number of CD3 and CD8 positive cells was counted by Image-Pro Plus 6.0 software.

### Statistical Analysis

Statistical analyses were performed with GraphPad Prism 8.0 software (San Diego, United States). Quantitative data were normally distributed and presented as mean ± SD. Statistically significant differences (*p* < 0.05) were examined using Student’s *t*-test or one-way ANOVA.

## Results

### PZH Inhibits the Proliferation of CRC Cells *in vitro*

**FIGURE 1:**
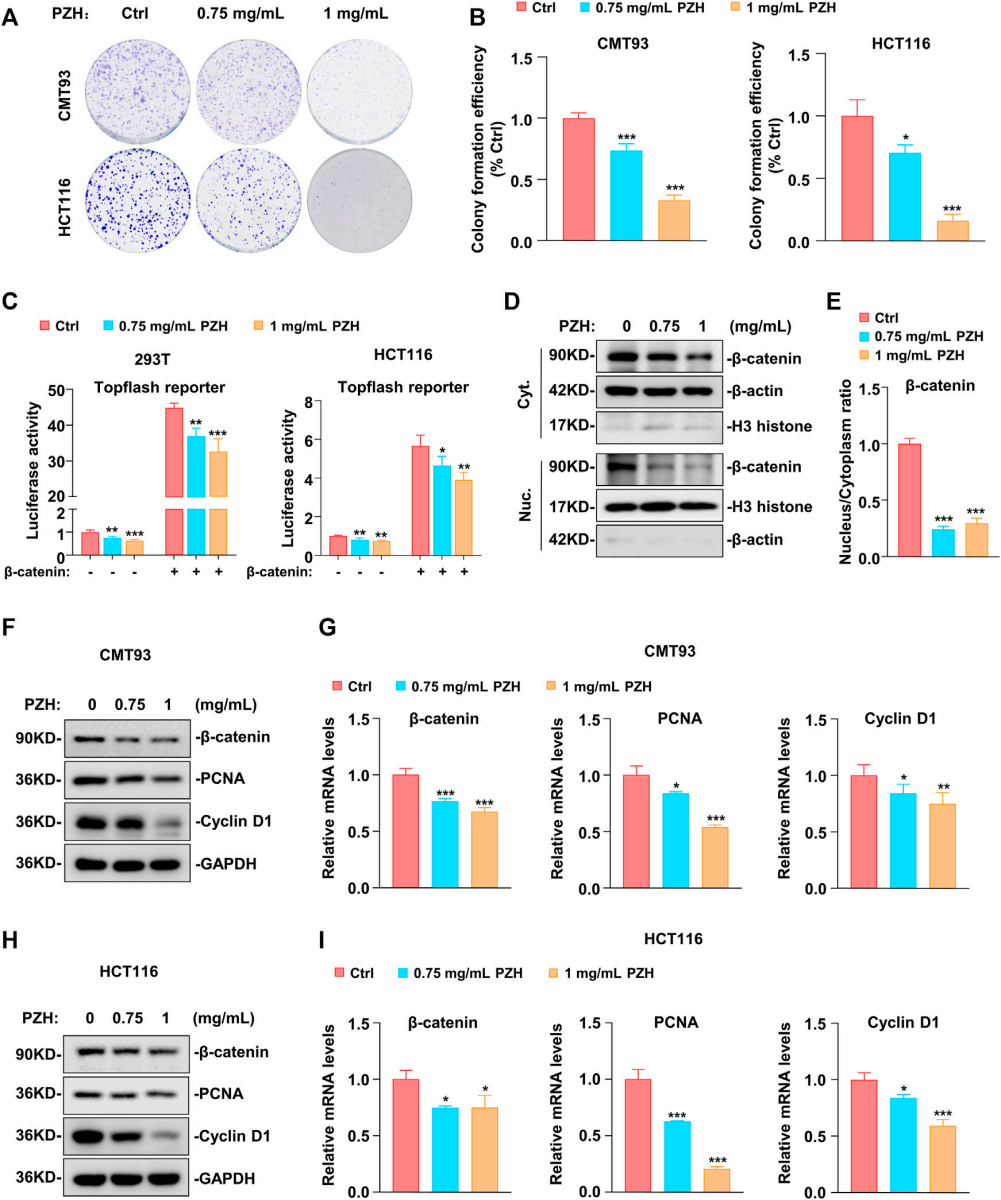
PZH suppressed the proliferation of CRC *in vitro* by inhibiting the expression of β-catenin, Cyclin D1 and PCNA. **(A–B)** Representative clonogenic analysis **(A)** and quantification of monoclonal number (**B**, *n* = 3 per group) of CMT93 and HCT116 cells. Tumor cells were treated with the indicated dose of PZH for 7 days, followed by methanol fixation and crystal violet staining, imaging, and the number of monoclonal cells was analyzed by Image Pro Plus 6.0 software. **(C)** PZH suppressed the Topflash reporter activity which was induced by β-catenin in 293T and HCT116 cells (*n* = 4 per group). **(D)** The cytoplasmic and nuclear protein levels of β-catenin were attenuated in PZH treated HCT116 cells. **(E)** PZH dramatically reduced the nuclear-cytoplasmic ratio of β-catenin (*n* = 3 per group). **(F–I)** The protein **(F,H)** and mRNA (**G,I**, *n* = 3-4 per group) levels of β-catenin, Cyclin D1 and PCNA were attenuated in PZH treated CMT93 and HCT116 cells. Tumor cells were treated with the indicated dose of PZH for 24h, followed by Western blot or qPCR analysis. The results are representative of one of three experiments. Data are presented as means ± SD, *p < 0.05, **p < 0.01, ***p < 0.001 based on one-way ANOVA or two-sided Student’s *t* test.

To investigate the effect of PZH on the proliferation of CRC cells, different doses of PZH were used to treat CMT93 and HCT116 cells. The results showed that PZH could inhibit the number and size of CRC cell colonies *in vitro* in a dose-dependent manner (Figures 1A,B), indicating that PZH could inhibit the proliferation of CRC cells *in vitro*.

Because abnormal Wnt signaling is a key promoter of colorectal tumorigenesis, Topflash, an established Wnt signaling reporter, was employed to determine whether PZH could suppress the activation of Wnt signaling. As shown in Figure 1C, PZH suppressed the basal Topflash reporter activity in 293T and HCT116 cells, implicating that PZH can suppress the activation of Wnt signaling. β-catenin, a key factor of the Wnt pathway, is mutated in up to 90% of CRC patients, resulting in abnormal activation of Wnt signaling to promote the progression of CRC (MacDonald et al., 2009; White et al., 2012). Therefore, exogenous β-catenin and Topflash reporter were co-transfected into 293T and HCT116 cells to evaluate whether PZH suppressed the activation of Wnt signal by inhibiting β-catenin. As shown in Figure 1C, β-catenin indeed significantly elevated the activity of Topflash reporter, but these activations were attenuated in PZH-treated cells. The data manifested that PZH may suppress Wnt signaling by inhibiting the transcriptional activity of β-catenin. In depth, we found that the protein levels of β-catenin in cytoplasm and nucleus were decreased in PZH-treated HCT116 cells (Figure 1D); and the nuclear-cytoplasmic ratio of β-catenin was reduced more dramatically (Figure 1E). Furthermore, we found that the protein and mRNA levels of β-catenin were attenuated in the whole cells (Figures 1F–I). These results demonstrate that PZH suppresses the expression and nuclear translocation of β-catenin, which may be the mechanisms by which PZH reduces β-catenin transcriptional activity. Our previous study has proved that β-catenin promotes the development of CRC by promoting the transcription of a variety of oncogenes, such as Cyclin D1 and PCNA (Peng et al., 2019). As expected, we found that PZH suppressed the protein and mRNA levels of Cyclin D1 and PCNA in CMT93 and HCT116 cells (Figures 1F–I).

Collectively, these results suggest that PZH inhibits the proliferation of CRC cells at least in part through suppressing the expression and nuclear translocation of β-catenin to down-regulate its target genes such as Cyclin D1 and PCNA, which are involved in cell proliferation.

### PZH Suppresses the Xenograft Tumor Growth in Mice

**FIGURE 2:**
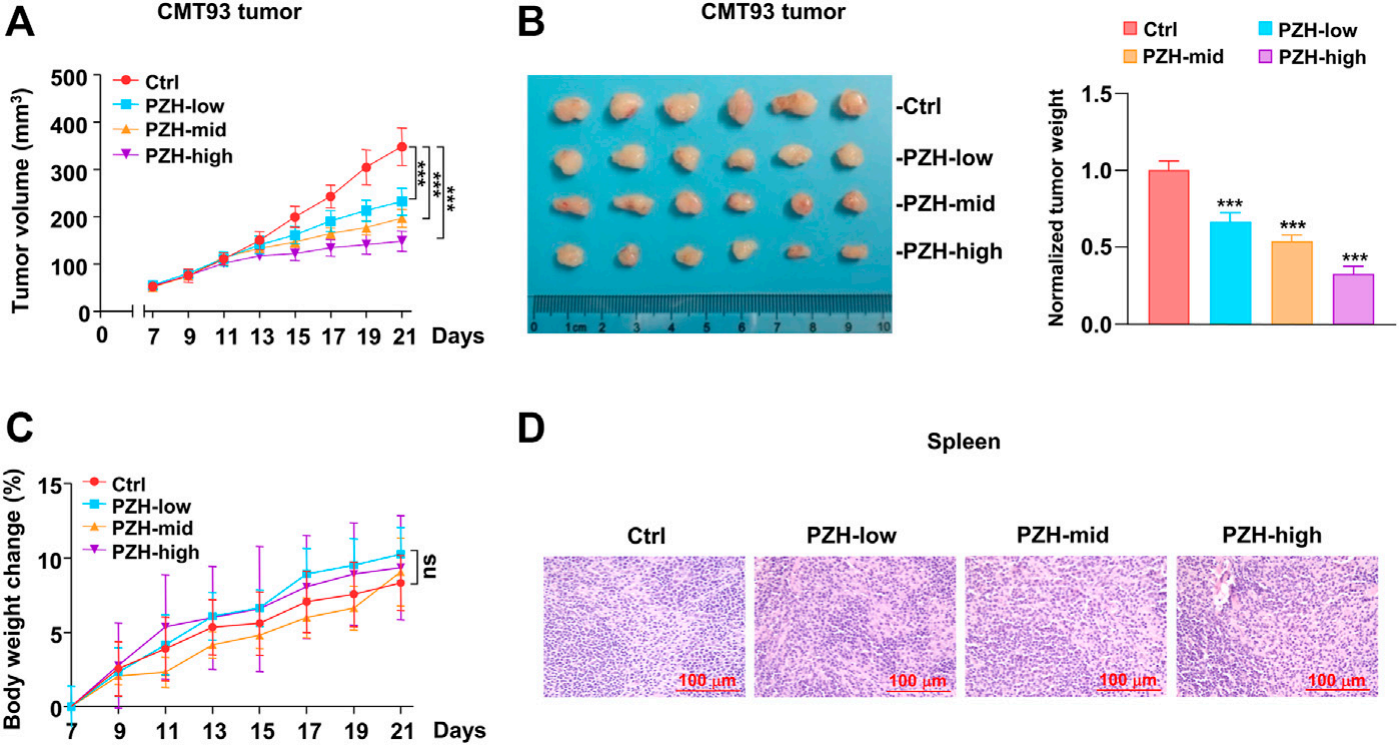
PZH stalled the CMT93 xenograft tumor growth *in vivo*. **(A–B)** The growth **(A)**, size and weight **(B)** of CMT93 xenograft tumor were attenuated in PZH treated mice compared with Ctrl group at day 21 of treatment (*n* = 6 per group): control and indicated dose of PZH (low: 0.078 g/kg; medium: 0.234 g/kg; high: 0.672 g/kg). Tumor weights were normalized with the mean value of Ctrl group. **(C)** PZH had no significant effect on the body weight of mice (*n* = 6 per group). **(D)** PZH had no obvious damage to the spleen of mice. The results are representative of one of three experiments. Data are presented as means ± SD, ^ns^ *p*>0.05, ***p < 0.001 based on one-way ANOVA.

As noted before, we have shown that PZH attenuated the proliferation of CRC cells *in vitro*. However, the effect of PZH on CRC growth *in vivo* is still unclear. Therefore, the xenograft tumor model was employed to investigate whether PZH has an inhibitory effect on CRC *in vivo*. CMT93 cells were inoculated into C57BL/6 mice, and the tumor growth was monitored. Seven days after inoculation, the mice were intragastrically administered with different amounts of PZH. The results showed that all three doses of PZH could suppress the xenograft tumor growth of CMT93 bearing mice (Figure 2A), and the tumor size and weight were also decreased in PZH-treated groups compared to Ctrl group in a dose-dependent manner (Figure 2B). We recorded the weight changes of mice during PZH treatment and found that PZH showed marginal effect on the weight of mice compared with the Ctrl group (Figure 2C). Spleen is one of the sites of lymphocyte maturation. To investigate whether PZH modulates the PD-1 expression or affects the activation of T cells, PMA/Ionomycin activated mouse lymphocyte cells were employed. We found that PZH had a marginal effect on the NFATC1 and PD-1 expression (Supplementary Figure S1), indicating that PZH had no effect on T cell activation. Again, the H&E results also showed that PZH had no obvious damage to the spleen of mice (Figure 2D). These results suggest that PZH has no toxicity to mice, especially to the immune function of mice.

**FIGURE 3:**
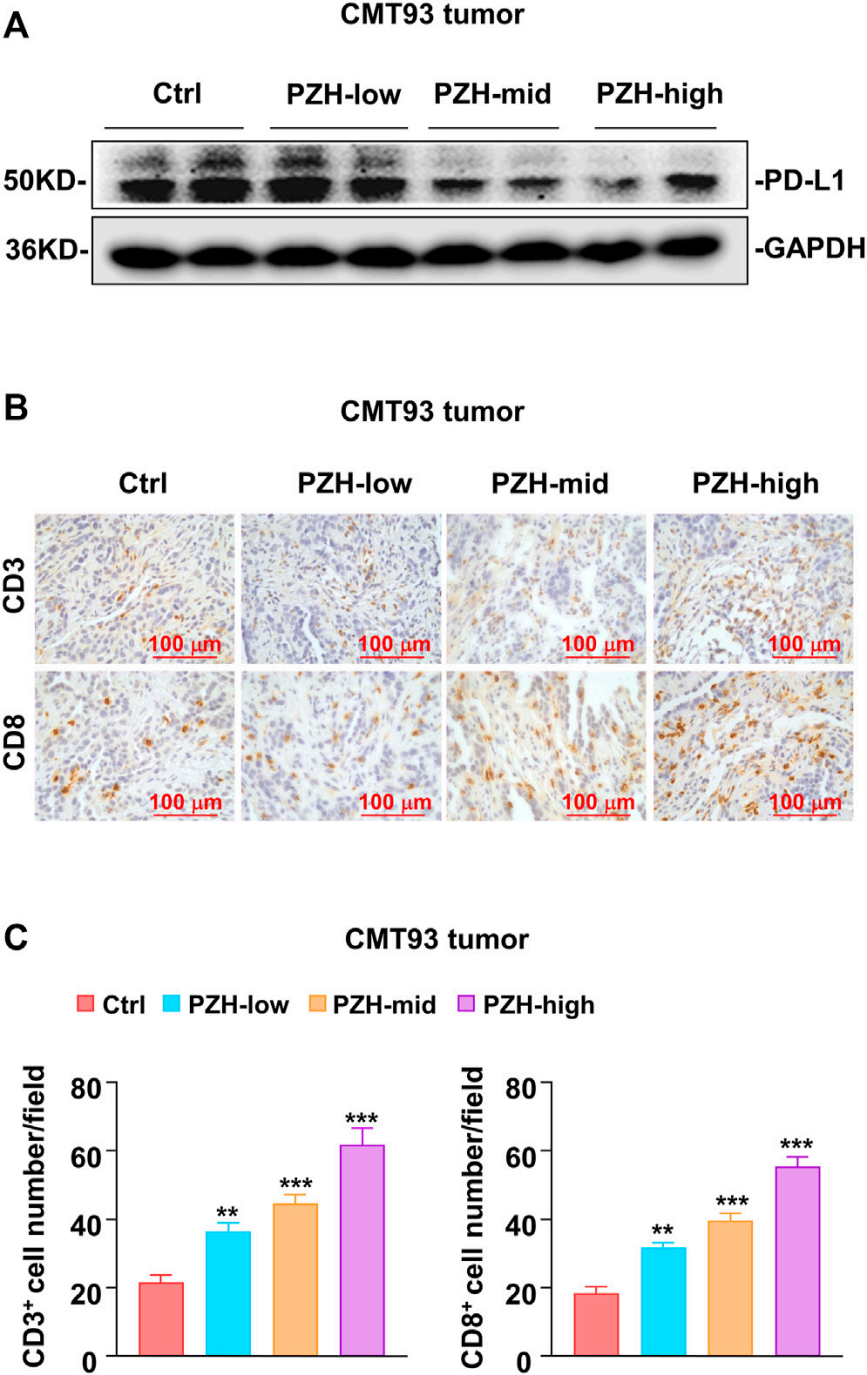
PZH elevated the infiltration of CD8^+^ T cells in tumor tissues. **(A)** PD-L1 protein levels were attenuated in the tumor tissues with indicated dose PZH treatment as described in **(** Figures 2A,B **)**. **(B–C)** Tumor-infiltrating CD3^+^ and CD8^+^ T cells were elevated in PZH-treated CMT93 tumor-bearing mice (*n* = 6 per group). CD3 and CD8 staining were performed by immunohistochemical analysis, and the number of positive cells was analyzed by Image Pro Plus 6.0 software. The results are representative of one of three experiments. Data are presented as means ± SD, **p < 0.01, ***p < 0.001 based on one-way ANOVA.

PD-L1 is one of the important immunosuppressive molecules on the surface of tumor cells, which can inhibit the activation and effector functions of CD8^+^ T cells by binding to PD-1 receptor. Intriguingly, we found that the protein levels of PD-L1 were decreased in the tumor tissues of PZH-treated mice (Figure 3A). Considering that tumor cells frequently escape T cell surveillance via over-expressing inhibitory molecule PD-L1, we speculated that suppression of PD-L1 by PZH can inhibit tumor immune escape. Therefore, the CD3 and CD8 staining was performed to evaluate T cell profiles of the tumor-infiltrating immune cells in CMT93 xenograft tumors. We observed much more CD3^+^ and CD8^+^ T cells infiltrated in the tumor tissues of PZH-treated mice than that of Ctrl tumors, especially in the high-dose of PZH groups (Figures 3B,C), indicating that PZH treatment can elevate the CD8^+^ T cell infiltration in the tumor tissues of CMT93 bearing mice. In conclusion, these results suggest that PZH relieves the immunosuppression of CD8^+^ T cells by suppressing PD-L1 expression in tumor cells, which may be one of the mechanisms for the antitumor effect of PZH.

### PZH Decreases PD-L1 Expression via Inhibiting the Basal STAT3-IRF1 Signaling

**FIGURE 4:**
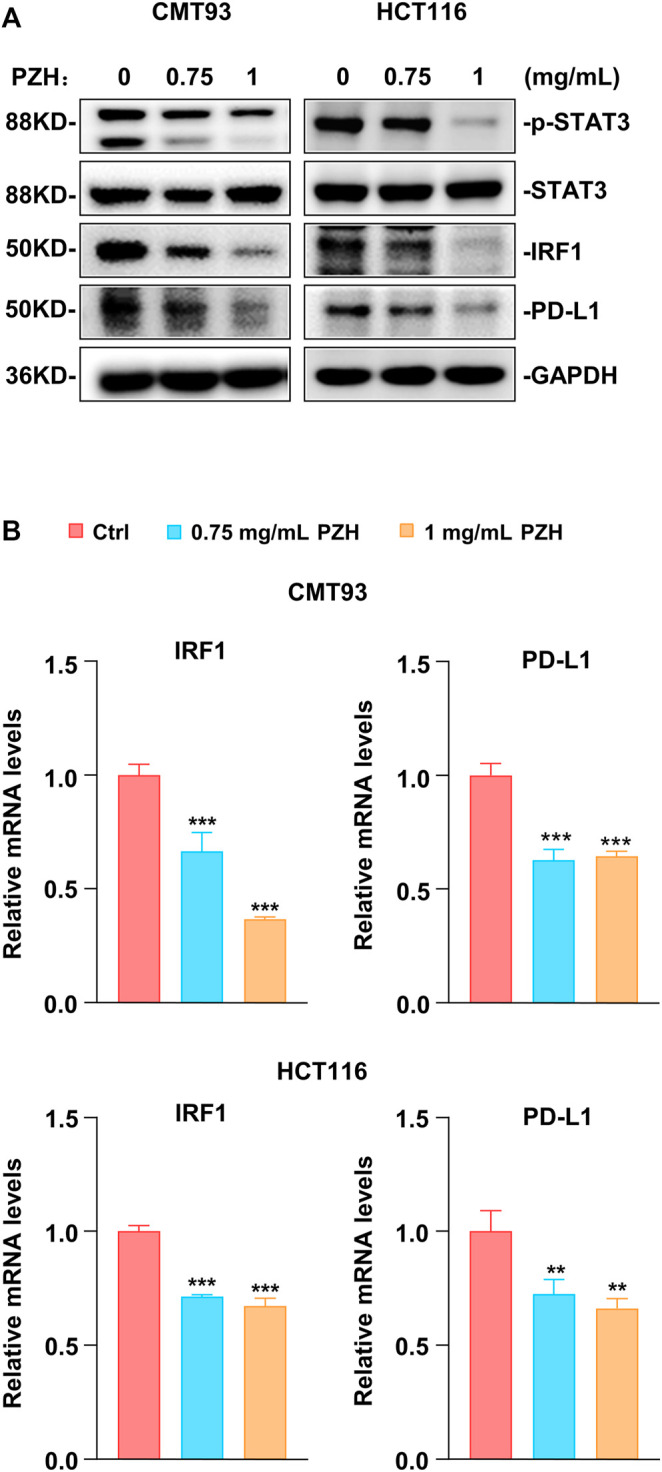
PZH suppressed the basal STAT3-IRF1 signaling to down-regulate PD-L1 expression. **(A)** The protein levels of p-STAT3, IRF1 and PD-L1 were dramatically attenuated in PZH treated CMT93 and HCT116 cells. **(B)** PZH treatment suppressed the mRNA expression of IRF1 and PD-L1 in CMT93 and HCT116 cells (*n* = 3 per group). The results are representative of one of three experiments. Data are presented as means ± SD, **p < 0.01, ***p < 0.001 based on one-way ANOVA.

Previous studies have shown that PZH inhibits the proliferation of CRC cells via suppressing the STAT3 signaling (Shen et al., 2012; Zhuang et al., 2012). STAT3 is one of the major transcriptional regulators of PD-L1; and IRF1, the downstream target gene of STAT3, also plays a key role in PD-L1 transcriptional regulation (Garcia-Diaz et al., 2017; Mimura et al., 2018). To investigate whether PZH affects PD-L1 expression through the STAT3-IRF1 pathway, the qPCR and Western blot were employed to detect the mRNA and protein expressions of genes involved in this pathway. Western blot results showed that PZH treatment decreased the protein levels of p-STAT3, IRF1, and PD-L1 compared to Ctrl group in CMT93 and HCT116 cells, while the protein levels of STAT3 were not changed in PZH-treated groups (Figure 4A). We also found that the mRNA levels of IRF1 and PD-L1 were decreased in PZH treatment CMT93 and HCT116 cells (Figure 4B), indicating that the transcriptional regulation of PD-L1 was suppressed by PZH treatment. These results suggest that PZH decreases the transcription of PD-L1 by inhibiting STAT3-IRF1 signaling.

### PZH Suppresses IFNγ-Induced Activation of STAT3-IRF1 Signaling and Up-Regulation of PD-L1

**FIGURE 5:**
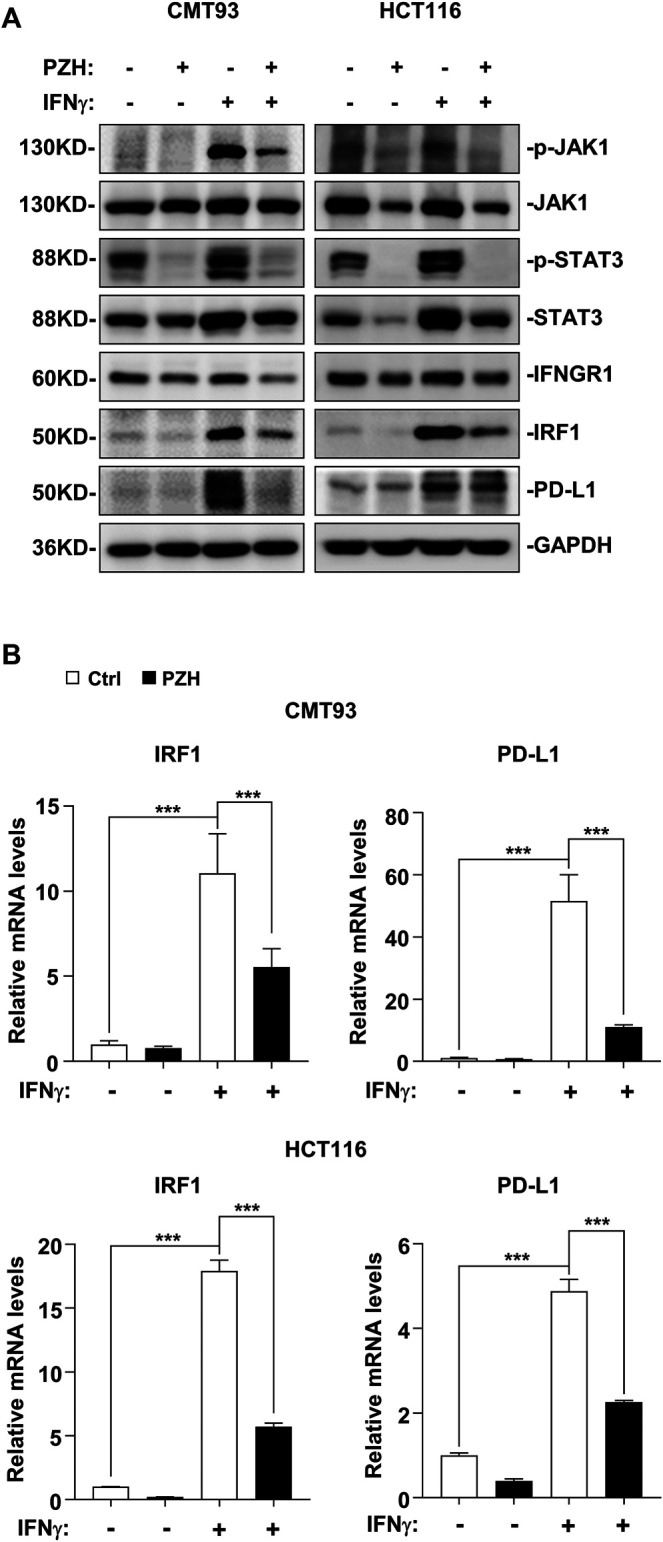
PZH suppressed IFNγ stimulation signal and PD-L1 up-regulation via inhibiting the IFNGR1-JAK1-STAT3-IRF1 signaling. **(A)** IFNγ-induced p-JAK1, p-STAT3, IRF1, and PD-L1 protein levels were attenuated in PZH treated CMT93 and HCT116 cells, as well as the basal IFNGR1 protein level. **(B)** The mRNA levels of IRF1 and PD-L1 induced by IFNγ were decreased in PZH treated CMT93 and HCT116 cells (*n* = 3–4 per group). The results are representative of one of three experiments. Data are presented as means ± SD, ***p < 0.001 based on one-way ANOVA.

IFNγ released by immune cells can trigger cancer immunosuppression against CD8^+^ T cell activation and effector function via activating JAK-STAT signal to induce the up-regulation of PD-L1 (Garcia-Diaz et al., 2017). PZH could suppress the basal STAT3-IRF1 signal in CRC cells (Figure 4), whether PZH also has an effect on IFNγ-induced activation of STAT3-IRF1 signaling remains unclear. Therefore, IFNγ-activated CRC cells were employed to evaluate the suppressive effect of PZH on activation of STAT3-IRF1 signaling by IFNγ. Upon IFNγ treatment, the protein levels of p-JAK1 and p-STAT3 as well as the protein and mRNA levels of IRF1 and PD-L1 were elevated significantly, but these inductions were attenuated by PZH treatment (Figures 5A,B). IFNγ signaling is transmitted through its receptor, i.e. IFNGR1, a high IFNγ affinity receptor chain (Lin et al., 2017; Walter, 2020). We found that PZH treatment reduced IFNGR1 protein levels (Figure 5A), suggesting that PZH attenuates IFNγ signaling at least in part through the inhibition of IFNGR1. These results suggest that PZH suppresses IFNγ-induced PD-L1 up-regulation via inhibiting the IFNGR1-JAK1-STAT3-IRF1 signaling.

### PZH Pharmacologically Elevates the Antitumor Efficacy of anti-PD-1/PD-L1 Immunotherapy

**FIGURE 6:**
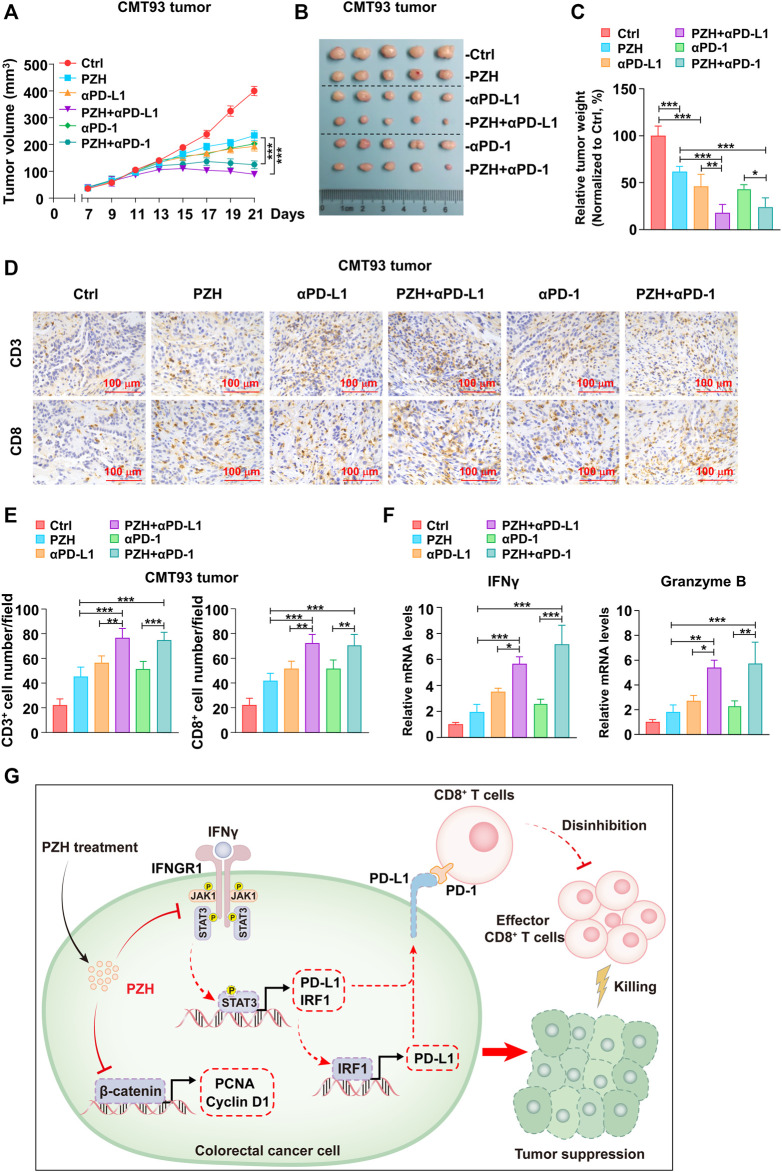
PZH synergistically promoted the antitumor efficacy of anti-PD-1/PD-L1 immunotherapy. **(A–B)** The tumor growth **(A)** and size **(B)** of CMT93 bearing mice were attenuated in the combination groups compared with the monotherapy groups at day 21 of treatment (*n* = 5 per group): IgG control, PZH (0.234 g/kg), PD-L1 antibody (100 μg), PZH + PD-L1 antibody combination group (PZH 0.234 g/kg and PD-L1 antibody 100 μg), PD-1 antibody (100 μg) and PZH + PD-1 antibody combination group (PZH 0.234 g/kg and PD-1 antibody 100 μg). **(C)** The treatment efficiency of combination group was higher than that of monotherapy with either agent (*n* = 5 per group). The treatment efficiency was quantified by tumor weight and normalized to the Ctrl group. **(D–E)** The number of tumor-infiltrating CD3^+^ and CD8^+^ cells was elevated in the combination groups with indicated treatments (*n* = 5 per group) as described in **(A–B)**. CD3 and CD8 staining were performed by immunohistochemical analysis, and the number of positive cells was analyzed by Image Pro Plus 6.0 software. **(F)** The mRNA levels of IFNγ and Granzyme B were up-regulated in the combination groups with indicated treatments (*n* = 3 per group) as described in **(A–B)**. The results are representative of one of three experiments. Data are presented as means ± SD, *p < 0.05, **p < 0.01, ***p < 0.001 based on one-way ANOVA.

Anti-PD-1/PD-L1 immunotherapy can attenuate the immunosuppression against T cell surveillance in tumor patients; however, it hardly eliminates the resistant tumor cells, which can evade the clearance of the immune system by up-regulating PD-L1 expression (Vesely et al., 2011; Oliveira et al., 2019). There are a considerable number of tumor patients who gradually lose sensitivity to PD-1/PD-L1 immunosuppressant therapy in clinical application. Therefore, how to improve the antitumor efficacy of PD-1/PD-L1 immunosuppressants is still a big challenge. In this study, the strategy of treating CMT93 tumor-bearing mice with PZH combined with PD-1/PD-L1 antibody was applied to evaluate the contribution of PZH in anti-PD-1/PD-L1 immunotherapy. The medium dose PZH and low dose PD-1/PD-L1 blocking antibody, as well as a combination of PZH and PD-1/PD-L1 antibody were used to treat mice inoculated with CMT93 cells. The results showed that PZH or PD-1/PD-L1 blocking antibody stalled the tumor growth and size (Figures 6A,B), whereas the combination group exhibited a more powerful tumor suppression compared to monotherapy with either agent (Figures 6A,B). The treatment efficiency was quantified by tumor weight and normalized to the Ctrl group, and we found that the combination group also had a higher treatment efficiency (Figure 6C).

Again, the T cell profiles of the tumor-infiltrating immune cells were evaluated by the CD3 and CD8 staining experiment in xenograft tumor tissues, and we found that the number of tumor-infiltrating CD3^+^ and CD8^+^ T cells were elevated in two combination groups compared with monotherapy group with either agent (Figures 6D,E). Consistently, the mRNA levels of IFNγ and Granzyme B, representing the activation and effector function of CD8^+^ T cells, were also higher in the combined groups than that in the monotherapy groups (Figure 6F). These results suggest that PZH synergistically promotes the infiltration and effector function of CD8^+^ T cells in tumor tissues during anti-PD-1/PD-L1 immunotherapy.

## Discussion

In this study, we demonstrated that PZH suppressed the proliferation of CRC cells *in vitro* and *in vivo*; and we also found that PZH attenuated CRC cell immunosuppression against CD8^+^ T cell surveillance via inhibiting the expression of PD-L1. The potential mechanism model was proposed (Figure 6G). Firstly, PZH suppressed CRC progression via inhibiting the expression and nuclear translocation of β-catenin, as well as the expression of proliferation-associated oncogenes, such as PCNA and Cyclin D1. Secondly, PZH attenuated the immune escape ability of CRC cells via inhibiting the IFNGR1-JAK1-STAT3-IRF1 signaling to down-regulate the level of PD-L1, which relieving the repression of CRC cells dependent PD-L1 inhibitory molecules on CD8^+^ T cells; these regenerated CD8^+^ T cells activated at the tumor site and exerted antitumor function.

The research to reveal the antitumor activity of PZH has been ongoing, and the potential antitumor mechanism of PZH is a pending mystery. It has been reported that PZH has certain antitumor efficacy in CRC, osteosarcoma, liver cancer and other tumors; however, the underlying mechanism is still obscure, although some potential mechanisms have been partially studied (Shen et al., 2013; Lin et al., 2015; Ren et al., 2015; Fu et al., 2017). Our study highlights the important roles of PZH in suppressing CRC proliferation by inhibiting the expression of PCNA and Cyclin D1 induced by β-catenin signaling. More importantly, we elucidated the potential role of PZH in mediating CRC immune escape and in synergistically enhancing anti-PD-1/PD-L1 immunotherapy, which depends on PZH attenuating PD-L1 expression via suppressing IFNGR1-JAK1-STAT3-IRF1 signaling. Shen et al. reported that PZH suppresses STAT3 activation by up-regulating SOCS3 expression, a negative regulator of STAT3 (Shen et al., 2012). In our research, we found that PZH treatment for a short period (24 h) had no obvious effect on the expression of STAT3 in CRC cells, but it dramatically suppressed the level of p-STAT3 (Figure 4A); while long-term treatment of PZH indeed attenuated the level of STAT3 (Figure 5A). Combined with the evidence that PZH suppressed the upstream receptor (IFNGR1) expression and kinase activation (JAK1) required for STAT3 activation, we speculated that PZH preferentially inhibited the phosphorylation of STAT3.

It is well known that the combination of multiple drugs in the treatment of cancer tends to have better results than that of monotherapy. PZH and its active ingredients have been widely used in a variety of diseases, achieving remarkable results in the field of tumor treatment with a combination of multiple drugs (Huang et al., 2019). PZH combined with routine radiotherapy or chemotherapy achieved a lower exacerbation rate in primary or advanced liver patients compared with monotherapy (Huang et al., 2019). PD-1/PD-L1 blocking antibody indeed elevates the effector function of CD8^+^ T cells in the short term, while activated effector T cells release a large amount of IFNγ to kill tumor cells, leading tumor cells to evade the surveillance of immune system through the inherent resistance mechanisms (Vesely et al., 2011; Garcia-Diaz et al., 2017). These surviving tumor cell populations are characterized by higher levels of PD-L1 and maintain a quantitative accumulation in the long term. This may be one of the important reasons why a considerable number of tumor patients gradually lose sensitivity to anti-PD-1/PD-L1 immunotherapy in clinical application. Intriguingly, PZH could suppress the IFNγ triggered tumor cell resistance via downregulating PD-L1 expression (Figure 5A), which improves the opportunity for PZH inhibited CRC immune escape. Importantly, our study revealed that PZH synergistically elevates the antitumor efficacy of anti-PD-1/PD-L1 immunotherapy, which provides powerful evidence for the immunotherapeutic adjuvant effect of PZH.

PZH has not been widely used in clinical anti-tumor treatment, while our study is only limited to verifying the inhibitory effect of PZH on the CRC progression and immune escape, as well as the synergistic enhancement effect of PZH on immunotherapy at the cellular level and animal models. We believe that with the in-depth development of PZH in clinical application, the clear anti-tumor mechanism will be clarified.

## Data Availability

The raw data supporting the conclusion of this article will be made available by the authors, without undue reservation.
