# Supplementary figures and images for: Traditional Chinese Medicine Pien-Tze-Huang Inhibits Colorectal Cancer Growth and Immune Evasion by Reducing β-catenin Transcriptional Activity and PD-L1 Expression
Source: Front Pharmacol. 2022 Feb 3;13:828440. doi: 10.3389/fphar.2022.828440 (PMC8850789; doi:10.3389/fphar.2022.828440)

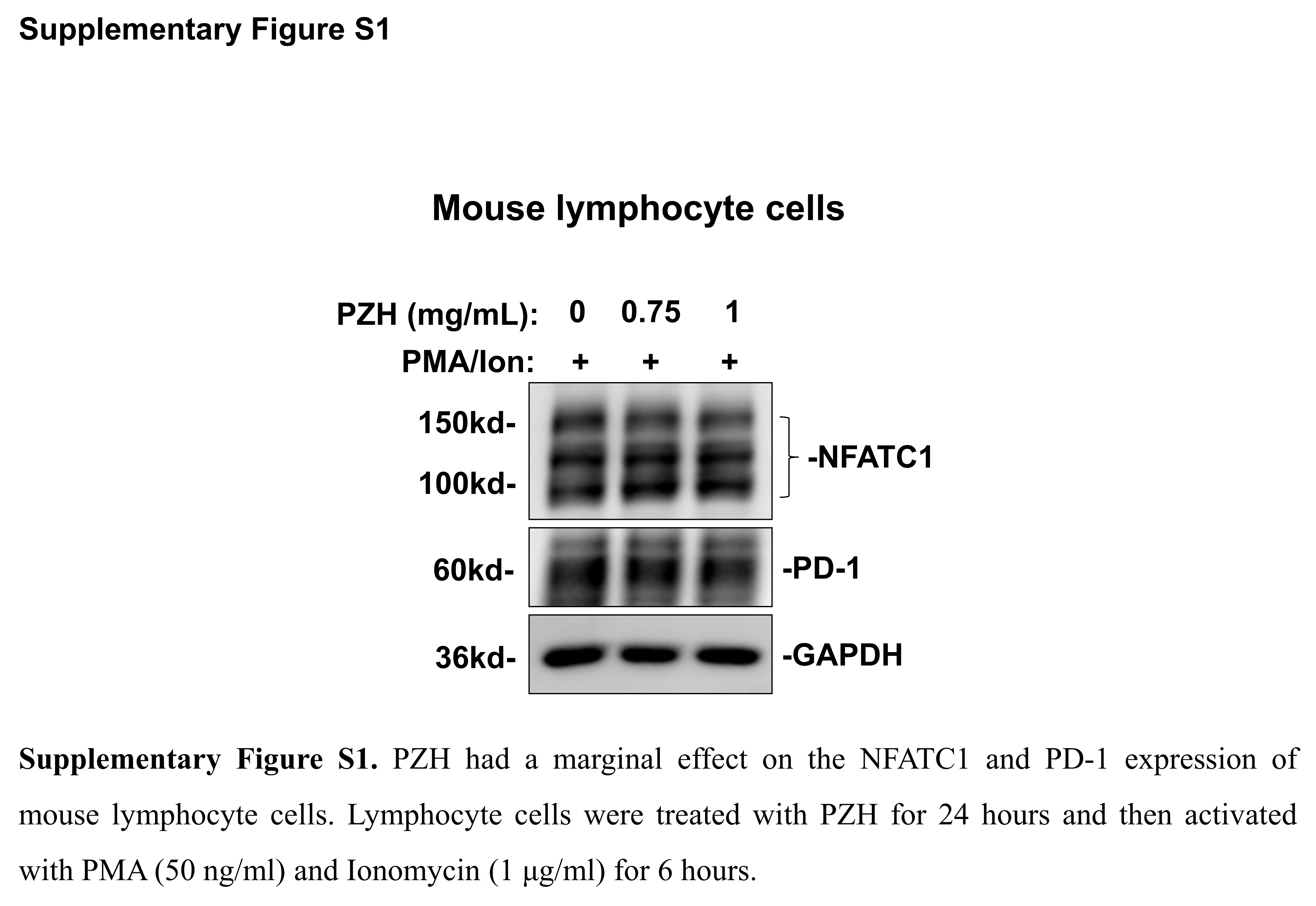

Supplement: Supplementary file 1 [file Image1.TIF]
